# Broadening Our Understanding Type 1 Diabetes Heterogeneity by Exploring Effects of Race/Ethnicity on Disease Trajectory

**DOI:** 10.1210/clinem/dgaa375

**Published:** 2020-06-15

**Authors:** Martin J Hessner, Susanne M Cabrera

**Affiliations:** 1 The Max McGee Diabetes Research Center, Children’s Research Institute of Children’s Wisconsin, Milwaukee, Wisconsin; 2 Department of Pediatrics, Medical College of Wisconsin, Milwaukee, Wisconsin

There is increasing recognition of heterogeneous pathogenic mechanisms underlying the immune mediated beta cell destruction that characterizes type 1 diabetes (T1D). Certainly, disease heterogeneity has long been clinically observed in the wide age range at diagnosis, variable insulin requirements, and differences in susceptibility to acute and chronic complications. Much of this may be attributed to the broad variability in residual beta-cell function at diagnosis and varying rates of functional beta-cell decline over time. Heterogeneity in beta-cell loss not only complicates clinical care but also confounds the use of beta-cell function, as measured by stimulated C-peptide during a standardized mixed meal tolerance test, as the primary (and clinically relevant) outcome measure in disease-modifying clinical trials that aim to preserve beta-cell function (1).

While trials of disease-modifying therapies have largely been unsuccessful in sustaining beta-cell preservation, further analyses of some interventions have defined distinct responder and nonresponder subgroups. Responsiveness to therapy has been attributed to unique genetic, immune, metabolic, and phenotypic features (1,2); for example, a plasma-induced transcription assay showed that subjects with high baseline innate inflammatory bias had more rapid C-peptide decline but also experienced the greatest therapeutic response to CTLA4-Ig (2). The heterogeneity in treatment responsiveness over many trials and increased elucidation of various mediating factors, led Battaglia et al to outline the need for an embrace of T1D as a constellation of distinct and heterogeneous “endotypes” (3). Endotypes are defined as disease subtypes with (distinct functional or pathobiological mechanisms) that tract to clinical phenotypes and therapeutic response. As endotypes are identified and defined by their pathobiological signatures, clinical trials would evolve to match subjects to therapies most likely to target their most predominant and active disease pathways, thereby increasing the benefit-to-risk ratio and shortening study durations.

Recognition of T1D heterogeneity also suggests that knowledge of disease mechanisms, natural history, and/or response to treatment gleaned from study of 1 endotype cannot be readily generalized to other endotypes or to the entire patient population. As such, findings from studies comprised almost exclusively of non-Hispanic White (NHW) subjects may not be generalizable to racial/ethnic minorities. This is especially important if race/ethnicity confers unique disease mechanisms. Indeed, ethnicity is known to affect T1D natural history in that progression from single to multiple islet autoantibodies is less common in Hispanics than NHW (4). Despite that protective finding, obese/overweight Hispanic youth with multiple autoantibodies have a much higher T1D risk than matched NHW (hazard ratio = 3.8 vs 1.36) (4). Further assessment of the role race/ethnicity plays in T1D outcomes is critical given the disproportional increases in T1D incidence experienced by racial/ethnic minorities in recent years with an annual increase of 4.2% in Hispanics versus 1.2% in NHW (5). In 2012, the SEARCH study reported 27, 19, and 14.8 new cases per 10 000 in NHW, non-Hispanic Blacks, and Hispanic American youth, respectively (5), reflecting T1D impact across the race/ethnicity spectrum. Subject enrollment into clinical trials should parallel these epidemiological trends. Unfortunately, racial/ethnic minorities remain grossly underrepresented in trials and there is a real risk that data gathered from mostly NHW has been inappropriately extrapolated across racial/ethnic groups.

In this issue of the *Journal of Clinical Endocrinology & Metabolism,* Tosur and colleagues explore the idea of race/ethnicity as contributing to unique disease endotypes by attempting to delineate how race/ethnicity impact the post-onset disease trajectory and rate of functional beta-cell decline in a cohort of subjects who had participated in 6 TrialNet new onset trials (6). Of the 624 participants, 87% self-reported NHW, and 8.7%, Hispanic. Other racial and ethnic groups could not be analyzed given their small numbers. Hispanics were younger and more overweight and had higher frequencies of ZnT8 autoantibody positivity and, concerningly, had higher rates of DKA at presentation than NHW (45% vs 25%). Hispanics had lower fasting glucose and C-peptide levels even after adjustment for body mass index, suggesting higher levels of insulin resistance and/or underestimation of adiposity by body mass index in Hispanics. The rate of functional beta-cell decline, as determined by serial mixed meal tolerance test over 3 years, was further assessed in 413 subjects (90.6% NHW; 9.4% Hispanic) who had participated in the placebo arm of 6 TrialNet trials and the experimental arm of 3 negative trials. Hispanics had higher stimulated C-peptide levels for 6 months but their rates of C-peptide decline subsequently paralleled those of NHW such that C-peptide levels were similar at 3 years post onset.

Despite differences in DKA rates and baseline metabolic and immunological characteristics, Hispanics and NHW had similar rates of beta-cell decline within 1 year. These data suggest it may be possible to generalize findings of beta cell preservation trials >1 year duration across NHW and Hispanics; however, caution is needed as participation in TrialNet trials requires consent into rigorous study protocols and baseline stimulated C-peptide ≥ 0.2 nmol/L (1). These requirements may exclude youth with lower socioeconomic status, language barriers, and more extensive beta-cell loss at diagnosis, factors that may disproportionately affect minority youth and skew Hispanic participants toward a subset not reflective of the ethnicity as a whole. Further, consolidation of Hispanics into a single ethnic group may erroneously oversimplify their significant genetic and geographical diversity and miss important distinctions.

Importantly, studies of this type ask us to consider if race/ethnicity health disparities in T1D outcomes have any pathobiological underpinnings or whether social determinants of health are primarily or solely responsible. Minority youth have significantly worse glycemic control and higher rates of complications than NHW (7,8). When socioeconomic status is considered, Hispanics and NHW appear more similar (7,8); however, Blacks have persistently worse glycemic control and higher rates of diabetic ketoacidosis than NHW even after controlling for socioeconomic factors (7,8). Could this be due to more aggressive beta-cell loss or unique inflammatory disease mechanisms? Unfortunately, the small subject numbers precluded assessment of disease trajectory in Blacks by Tosur et al. For the same reason, analyses of response to disease-modifying therapies in TrialNet trials by ethnicity/race have not been possible. It remains unanswered to what extent mostly NHW-derived knowledge of T1D pathobiology matches that occurring in racial/ethnic minorities, but initial studies suggest racial/ethnic distinctions that preclude full generalizability and require more study (4,6).

Further elucidation of race/ethnicity-specific mechanisms of disease in the broader context of T1D endotypes holds the promise of developing targeted disease-modifying treatments, mitigating health disparities, and improving clinical care delivery. Central to this mission must be concerted commitment to increase minority youth study participation proportionate to their disease incidence and disease burden.

## Data Availability

Data sharing is not applicable to this article as no datasets were generated or analyzed during the current study.

## Additional Information

***Disclosures Summary:*** The authors have nothing to declare.
